# Body Mass Index and Outcomes After Transcatheter Aortic Valve Replacement: Insights From the Transpacific-TAVR Registry

**DOI:** 10.1016/j.jacasi.2025.06.012

**Published:** 2025-09-02

**Authors:** Eric P. Cantey, Ashraf Samhan, Abigail S. Baldridge, S. Chris Malaisrie, Charles J. Davidson, Alan C. Yeung, William F. Fearon, Do-Yoon Kang, Seung-Jung Park, Duk-Woo Park, James D. Flaherty

**Affiliations:** a Division of Cardiology, Department of Medicine, University of Michigan, Ann Arbor, Michigan, USA; b Bluhm Cardiovascular Institute, Northwestern University Feinberg School of Medicine, Chicago, Illinois, USA; c Section of Cardiology, Division of Medicine, Stanford University School of Medicine, Palo Alto, California, USA; d Division of Cardiology, Asan Medical Center, University of Ulsan College of Medicine, Seoul, South Korea

**Keywords:** aortic stenosis, body mass index, left ventricular remodeling, transcatheter aortic valve replacement

## Abstract

**Background:**

Whereas some studies suggest an "obesity paradox" with improved outcomes in obese patients following transcatheter aortic valve replacement (TAVR), the impact of pre-TAVR body mass index (BMI) and post-TAVR BMI changes on clinical and echocardiographic outcomes remains unclear.

**Objectives:**

This study sought to evaluate the influence of BMI at the time of TAVR and subsequent BMI changes on clinical and echocardiographic outcomes in patients undergoing TAVR.

**Methods:**

We included 1,339 patients with severe, native aortic stenosis from 2015 to 2019, stratified by BMI according to World Health Organization classifications, from an international registry. The primary outcome was overall survival, with secondary outcomes including short- and long-term survival, bleeding, vascular injury, stroke, and acute kidney injury. Descriptive statistics and time-to-event analyses were performed.

**Results:**

Underweight patients were older (n = 45; age 83.8 ± 6.6 years) compared to normal weight (n = 576; 81.5 ± 7.0 years), overweight (n = 438; age 81.0 ± 7.4 years), and obese (n = 280; age 77.4 ± 8.3 years; *P* < 0.001) patients. Underweight patients had the highest rates of chronic kidney disease (66.7%) and moderate or greater aortic regurgitation (28.9%). Obese patients had the highest rates of atherosclerotic cardiovascular disease risk factors. Over a median follow-up of 1.1 (Q1-Q3: 0.6-2.7) years, there were no significant differences between BMI groups (*P* = 0.69). At 1-year follow-up, underweight patients showed improved left ventricular remodeling and favorable TAVR hemodynamics.

**Conclusions:**

Pre-TAVR BMI did not significantly affect clinical outcomes in this diverse cohort, challenging the obesity paradox. However, underweight patients exhibited subtle improvements in left ventricular remodeling and valve hemodynamics post-TAVR, highlighting a nuanced role for BMI in recovery.

Transcatheter aortic valve replacement (TAVR) has rapidly evolved into an established treatment for patients with severe, symptomatic, trileaflet aortic stenosis, offering a viable alternative to surgical aortic valve replacement.^1^, ^2^, ^3^, ^4^, ^5^, ^6^ Advances in technical approaches have reduced the procedural complexity of TAVR, resulting in outcomes comparable to or better than those of surgical aortic valve replacement. Beyond these technical innovations, identifying preprocedural risk factors remains crucial for enhancing risk stratification. Aortic stenosis represents a growing health concern worldwide, including in the Asia-Pacific region, where its prevalence is increasing with the aging population. Therefore, understanding factors that influence TAVR outcomes in diverse populations is paramount.

The impact of body mass index (BMI) on post-TAVR outcomes is a subject of ongoing debate. Several studies have highlighted an “obesity paradox,” suggesting a protective effect of obesity on post-TAVR outcomes.^7^, ^8^, ^9^, ^10^ Reflecting this observation, the VARC-2 (Valve Academic Research Consortium-2) guidelines classify BMI <20 kg/m^2^ as a marker of frailty, which correlates with increased peri- and postprocedural risk.^11^ However, these studies primarily focus on Western populations with high obesity prevalence and are limited by short-term follow-up. Furthermore, the relationship between BMI and post-TAVR echocardiographic hemodynamics and the impact of BMI changes after TAVR on procedural outcomes remains unexplored.

This study aims to address these gaps by examining the influence of pre-TAVR BMI and post-TAVR BMI changes on clinical and echocardiographic outcomes, leveraging data from a multicenter, international registry.

## Methods

The TP (TransPacific)-TAVR registry is an international, multicenter cohort study involving consecutive patients who underwent TAVR for severe, symptomatic aortic stenosis. In 2019, investigators abstracted demographic, clinical, echocardiographic, procedural, and outcome variables from the electronic medical record of 3 clinical sites: Stanford University and Northwestern University (patients from June 2016 to November 2019) and Asan Medical Center (patients from January 2015 to November 2019). Each institution's multidisciplinary heart team assessed TAVR candidacy, conducted preprocedure planning, performed the TAVR procedures, monitored for postprocedural complications, and conducted routine follow-ups per established guidelines and local protocols.

All data were standardized into a common database model and merged into a centralized database maintained by the Clinical Research Coordinating Center (Cardiovascular Research Foundation). Each center’s Institutional Review Board approved the registry’s design and implementation. The Cardiovascular Research Foundation and the Asan Institute for Life Sciences and Corporate Relations partially funded the study but had no role in its design, data analysis, or manuscript preparation.

Subjects included in the analysis were those undergoing TAVR for native valve, severe aortic stenosis. Investigators calculated each subject's BMI (kg/m^2^) using anthropometric data collected during the pre-TAVR evaluation and categorized it into 4 groups based on the World Health Organization classification: underweight (<18.5 kg/m^2^); normal weight (≥18.5 and <25 kg/m^2^); overweight (≥25 and <30 kg/m^2^); and obese (≥30 kg/m^2^). Institutional radiologists and echocardiographers evaluated each study following established guidelines.^12^, ^13^, ^14^ All outcomes were adjudicated per VARC-2 criteria, with survival data obtained from electronic medical records and public death registries.^11^

The primary outcome was overall survival. Secondary clinical outcomes included 30-day and 1-year mortality, 30-day rehospitalization, major and life-threatening bleeding, major vascular injury, stroke, permanent pacemaker implantation, and stage 2 or 3 acute kidney injury. Among subjects with echocardiograms available at 1-year post-TAVR, investigators analyzed changes in the left ventricular mass index from pre-TAVR echocardiograms; trans-TAVR valve gradients; and rates of patient-prosthetic mismatch; TAVR valve stenosis; and greater-than-moderate perivalvular, mitral, and tricuspid regurgitation across BMI groups. Subgroup analyses included comparisons within Asian and non-Asian subjects. Statistical analyses included analysis of variance and Kruskal-Wallis test for continuous variables and chi-square or Fisher exact tests for categorical variables to assess differences between BMI groups. Differences in echocardiographic characteristics between groups from baseline to 1-year follow-up were evaluated through Kruskal-Wallis tests. Baseline covariates univariably associated with the obesity group at baseline at *P* ≤ 0.20 were evaluated for association with mortality at 30 days and 1 year. From the candidate pool, variables univariably associated with mortality at *P* ≤ 0.15 were selected for multivariable logistic regression modeling. Kaplan-Meier survival curves with log-rank tests were used for time-to-event analysis of the primary outcome. Statistical significance was defined as *P* < 0.05 with no adjustment for multiple comparisons. All analyses were performed using SAS software (version 9.4, SAS Institute) or R (version 4.0.3, R Foundation).

## Results

Of the 1,412 consecutive patients undergoing TAVR, 73 underwent valve-in-valve TAVR procedures. Thus, 1,339 subjects were included in the final analysis, of which 3.3% (45) were underweight, 43.0% (576) had a normal weight, 32.7% (439) were overweight, and 20.9% (280) were obese. Table 1 displays baseline characteristics of the BMI group. On average, underweight individuals were older (age 83.8 ± 6.6 years) compared to normal weight (age 81.5 ± 7.0 years), overweight (age 81.0 ± 7.4 years), and obese (age 77.4 ± 8.3 years; *P* < 0.001) patients. Underweight individuals were also more likely to be of Asian descent (24 of 45; 53.3%) compared to normal weight (341 of 576; 59.2%), overweight (169 of 438; 38.4%), and obese patients (28 of 280; 10%; *P* < 0.001). Underweight patients had a higher median Society of Thoracic Surgery–predicted risk of mortality (5.5; Q1-Q3: 3.7-7.8) compared to normal weight (4.0; Q1-Q3: 2.8-6.6), overweight (3.7; Q1-Q3: 2.6-5.8), and obese patients (4.0; Q1-Q3: 2.9-6.0; *P* < 0.001).

**Table 1:** Baseline Characteristics by BMI Group

	Underweight (n = 45)	Normal (n = 576)	Overweight (n = 438)	Obesity (n = 280)	Overall (N = 1,339)	*P* Value^a^
Age at TAVR, y	83.8 ± 6.6	81.5 ± 7.0	81.0 ± 7.4	77.4 ± 8.3	80.5 ±7.6	<0.001
Male	17 (37.8)	301 (52.3)	241 (55.0)	150 (53.6)	709 (52.9)	0.17
Race						<0.001
Non-Asian	21 (46.7)	235 (40.8)	270 (61.6)	252 (90.0)	778 (58.1)	
Asian	24 (53.3)	341 (59.2)	168 (38.4)	28 (10.0)	561 (41.9)	
BMI, kg/m^2^	17.0 ± 1.4	22.4 ± 1.7	27.3 ± 1.4	35.5 ± 5.9	26.6 ± 6.1	<0.001
Comorbidities						
Coronary artery disease	25 (55.6)	288 (50.0)	263 (60.0)	200 (71.4)	776 (58.0)	<0.001
Myocardial infarction	8 (17.8)	59 (10.2)	52 (11.9)	32 (11.4)	151 (11.3)	0.44
Percutaneous coronary intervention	13 (28.9)	167 (29.0)	130 (29.7)	83 (29.6)	393 (29.4)	0.99
Atrial fibrillation or flutter	11 (24.4)	132 (22.9)	128 (29.2)	107 (38.2)	378 (28.2)	<0.001
Peripheral arterial disease	6 (13.3)	72 (12.5)	67 (15.3)	68 (24.3)	213 (15.9)	<0.001
Stroke	3 (6.7)	66 (11.5)	57 (13.0)	25 (8.9)	151 (11.3)	0.28
Diabetes mellitus	14 (31.1)	218 (37.8)	177 (40.4)	149 (53.2)	558 (41.7)	<0.001
Diabetes mellitus on insulin	12 (26.7)	144 (25.0)	153 (34.9)	134 (47.9)	443 (33.1)	<0.001
Hypertension	34 (75.6)	489 (84.9)	384 (87.7)	250 (89.3)	1157 (86.4)	0.042
Hyperlipidemia	24 (53.3)	421 (73.1)	338 (77.2)	212 (75.7)	995 (74.3)	0.005
History of pacemaker	3 (6.7)	35 (6.1)	19 (4.3)	29 (10.4)	86 (6.4)	0.015
Bicuspid aortic valve	6 (13.3)	45 (7.8)	24 (5.5)	12 (4.3)	87 (6.5)	0.044
Chronic kidney disease	30 (66.7)	374 (64.9)	202 (46.1)	101 (36.1)	707 (52.8)	<0.001
End-stage renal disease on dialysis	0 (0)	28 (4.9)	11 (2.5)	11 (3.9)	50 (3.7)	0.13
NYHA functional class III or IV	23 (51.1)	230 (39.9)	172 (39.3)	152 (54.3)	577 (43.1)	<0.001
STS risk score, %	5.5 (3.7-7.8)	4.0 (2.8-6.6)	3.7 (2.6-5.8)	4.0 (2.9-6.0)	3.9 (2.8-6.2)	<0.001
Creatinine, mg/dL	0.9 (0.8-1.2)	1.0 (0.8-1.3)	1.0 (0.8-1.3)	1.1 (0.8-1.4)	1.0 (0.8-1.3)	0.008
Albumin, mg/dL	3.6 (3.2-3.8)	3.6 (3.3-3.9)	3.8 (3.5-4.0)	3.9 (3.7-4.2)	3.7 (3.4-4.0)	<0.001
Hemoglobin, g/dL	11.3 ± 1.6	11.7 ± 1.9	12.2 ± 1.9	12.2 ± 1.9	11.9 ± 1.9	<0.001
Procedural characteristics						
Transfemoral access	43 (95.6)	553 (96.0)	426 (97.3)	270 (96.4)	1292 (96.5)	0.73
Balloon-expandable valve	40 (88.9)	494 (85.8)	390 (89.0)	246 (87.9)	1170 (87.4)	0.46
General anesthesia	16 (35.6)	220 (38.2)	178 (40.6)	135 (48.2)	549 (41.0)	0.037

Values are mean ± SD, n (%), or median (Q1-Q3).

BMI = body mass index; STS = Society of Thoracic Surgery; TAVR = transcatheter aortic valve replacement.

a Differences across groups were compared by analysis of variance test, Kruskal-Wallis test, or chi-square test as appropriate.

Underweight individuals were more likely to have a bicuspid valve morphology (6 of 45; 13.3%) compared to normal weight (45 of 576; 7.8%), overweight (24 of 438; 5.5%), and obese (12 of 280; 4.3%; *P* = 0.044). Underweight individuals were similarly more likely to have pre-existing chronic kidney disease (CKD) (*P* < 0.001), lower median preprocedural albumin (*P* < 0.001), and lower hemoglobin levels (*P* < 0.001) compared to the other BMI groups.

Obese individuals exhibited the highest rates of cardiovascular risk factors that differed across groups, including diabetes (149 of 280; 53.2%; *P* < 0.001), hypertension (250 of 280; 89.3%; *P* = 0.042), and hyperlipidemia (212 of 280; 75.7%; *P* = 0.005). They also had the highest rates of cardiovascular disease, such as coronary artery disease (200 of 280; 71.4%; *P* < 0.001), peripheral arterial disease (68 of 280; 24.3%; *P* < 0.001), and atrial fibrillation/flutter (107 of 280; 38.2%; *P* < 0.001) compared to the other BMI groups.

Supplemental Table 1 compares preprocedural variables among Asian and non-Asian subjects. Whereas underweight individuals across both groups demonstrated increased age, higher Society of Thoracic Surgery–predicted risk of mortality, and higher diabetes prevalence, Asian underweight subjects showed more pronounced increases in CKD rates, lower rates of hypertension and hyperlipidemia, and lower preprocedural hemoglobin levels.

Table 2 outlines baseline echocardiographic characteristics by BMI group. Underweight individuals had the lowest median preprocedural left ventricular ejection fraction (58%; Q1-Q3: 47%-66%; *P* = 0.015), highest median left ventricular mass index (132 g/m^2^; Q1-Q3: 89-152 g/m^2^; *P* < 0.001), highest mean peak transaortic velocity (4.6 ± 0.9 m/s; *P* < 0.001), and lowest mean aortic valve area (0.59 ± 0.18 cm^2^; *P* < 0.001). They also exhibited the highest rates of eccentric hypertrophy (7 of 45; 22.6%; *P* < 0.001), moderate or greater aortic regurgitation (13 of 45; 28.9%; *P* < 0.001), and at least moderate or greater mitral regurgitation (14 of 45; 31.1%; *P* = 0.002) compared to the other BMI groups.

**Table 2:** Baseline Echocardiographic Characteristics by BMI Group

	Underweight (n = 45)	Normal (n = 576)	Overweight (n = 438)	Obesity (n = 280)	Overall (N = 1,339)	*P* Value^a^
Left ventricular ejection fraction, %	58 (47-66)	60 (50-65)	61 (55-65)	62 (55-66)	60 (53-66)	0.015
Left ventricular ejection fraction <40%	10 (22.2)	99 (17.2)	56 (12.8)	31 (11.1)	196 (14.6)	0.028
LVMI, g/m^2^	132 (89-152)	125 (103-150)	117 (100-136)	105 (85-129)	118 (98-143)	<0.001
Remodeling pattern						<0.001
Normal	1 (3.2)	32 (7.5)	28 (9.9)	20 (13.0)	81 (9.0)	
Concentric Hypertrophy	13 (41.9)	223 (52)	137 (48.2)	59 (38.3)	432 (48.1)	
Concentric remodeling	10 (32.3)	89 (20.7)	74 (26.1)	61 (39.6)	234 (26.1)	
Eccentric hypertrophy	7 (22.6)	85 (19.8)	45 (15.8)	14 (9.1)	151 (16.8)	
Peak velocity (m/s)	4.6 ± 0.9	4.6 ± 0.8	4.6 ± 0.8	4.3 ± 0.6	4.5 ± 0.8	<0.001
Mean gradient, mm Hg	47 (40-61)	48 (40-62)	47 (40-60)	45 (38-54)	47 (40-60)	<0.001
AVA, cm^2^	0.59 ± 0.18	0.63 ± 0.18	0.67 ± 0.18	0.75 ± 0.19	0.67 ± 0.19	<0.001
Low flow low gradient						0.28
None	32 (71.1)	430 (74.7)	322 (73.5)	201 (71.8)	985 (73.6)	
Classical	11 (24.4)	105 (18.2)	79 (18.0)	46 (16.4)	241 (18.0)	
Paradoxical	2 (4.4)	41 (7.1)	37 (8.4)	33 (11.8)	113 (8.4)	
≥ Moderate AR	13 (28.9)	89 (15.5)	45 (10.3)	21 (7.5)	168 (12.5)	<0.001
≥ Moderate MR	14 (31.1)	99 (17.2)	67 (15.3)	29 (10.4)	209 (15.6)	0.002
≥ Moderate TR	9 (20.0)	68 (11.8)	50 (11.4)	25 (8.9)	152 (11.4)	0.17

Values are mean ± SD, n (%), or median (Q1-Q3).

AR = atrial regurgitation; AVA = aortic valve area; LVMI - left ventricular mass index; MR = mitral regurgitation TR = tricuspid regurgitation; other abbreviations as in Table 1.

a Differences across groups were compared by analysis of variance test, Kruskal-Wallis test, or chi-square test as appropriate.

Supplemental Table 2 compares baseline echocardiographic characteristics between Asian and non-Asian cohorts. Non-Asian underweight subjects had the lowest median left ventricular ejection fraction (58%; Q1-Q3: 48%-66%; *P* < 0.001), smallest aortic valve areas (0.62 ± 0.21 cm^2^; *P* < 0.001), and highest rates of at least moderate aortic (6 of 21; 28.6%; *P* = 0.004), mitral (9 of 45; 42.9%; *P* < 0.001), and tricuspid regurgitation (7 of 45; 33.3%; *P* = 0.001) compared to the other non-Asian BMI groups. No significant differences in preprocedural echocardiographic variables were observed among Asian BMI groups.

**Figure 1:**
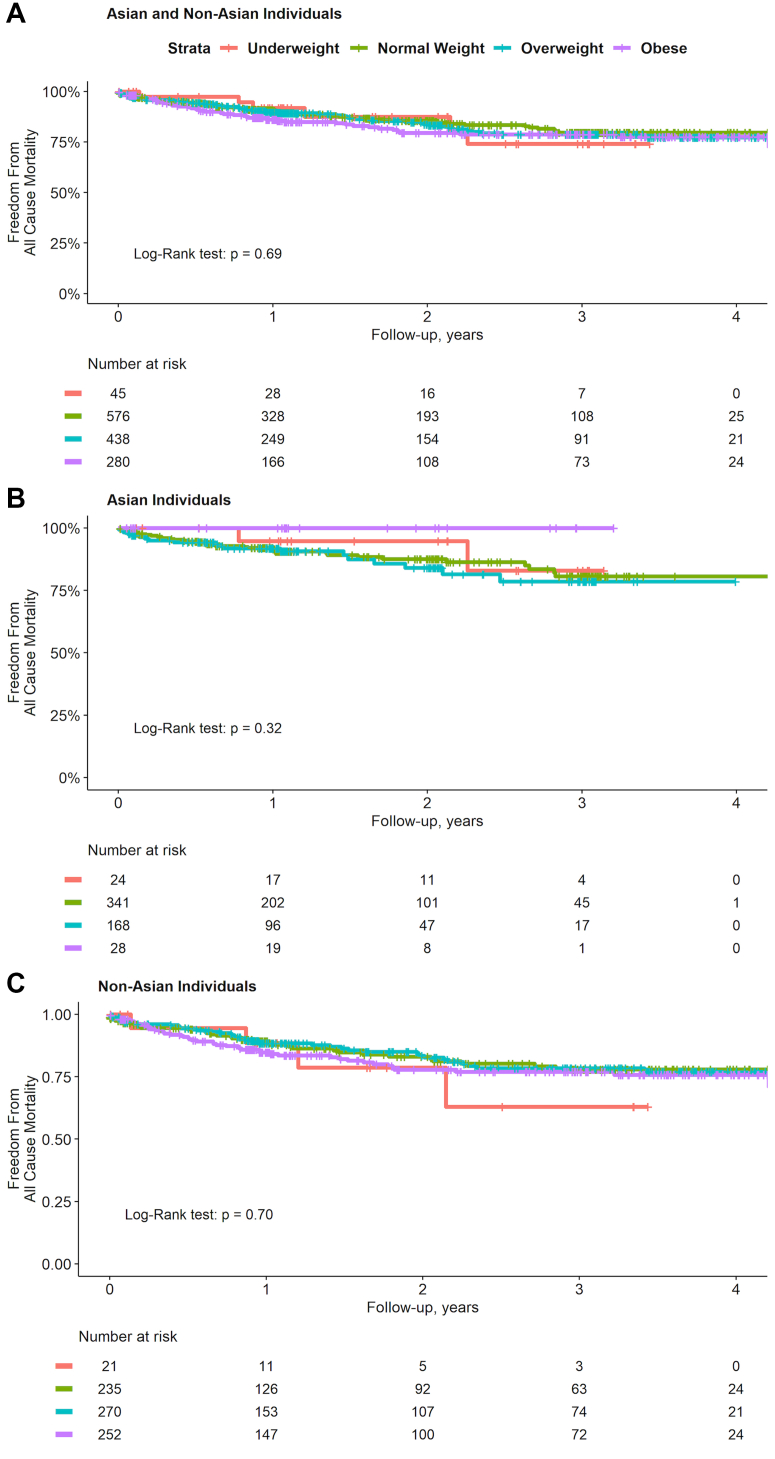
Kaplan-Meier Curves for All-Cause Mortality by BMI and Ethnicity Kaplan-Meier survival curves illustrating the freedom from all-cause mortality over a 4-year follow-up period, stratified by body mass index (BMI) category (underweight, normal weight, overweight, obese) for 3 groups: (A) Asian and non-Asian individuals combined, (B) Asian individuals only, and (C) non-Asian individuals only. The number of individuals at risk in each BMI category at different time points is provided below each respective plot. Log-rank *P* values are shown for each comparison, assessing the overall difference in survival across BMI categories within each group.

Figure 1 illustrates post-TAVR survival by BMI group, with a median follow-up of 1.1 years (Q1-Q3: 0.6-2.7 years). Overall survival was 76.6% (95% CI: 72.8%-80.7%) over 4 years of follow-up. No significant differences in overall survival were observed between BMI groups (log-rank *P* = 0.69) or within Asian and non-Asian subgroups by BMI group.

Table 3 outlines post-TAVR outcomes by BMI group. Normal-weight individuals had higher rates of 30-day major or life-threatening bleeding (67 of 491; 13.6%) compared to underweight (4 of 39; 10.3%), overweight (33 of 378; 8.7%), and obese (4 of 251; 1.6%; *P* < 0.001), with similar 1-year trends observed. Obese individuals had higher rates of 30-day pacemaker implantation (35 of 262; 13.4%) compared to underweight (4 of 42; 9.5%), normal weight (42 of 542; 7.7%), and overweight (51 of 490; 12.5%; *P* = 0.039) groups, with similar 1-year trends observed. Both results were attenuated after multivariable adjustment. No significant differences in mortality, rehospitalization, major vascular injury, stroke, or acute kidney injury at either 30 days or 1 year were observed between BMI groups, and results were attenuated when stratified by subpopulations (Supplemental Tables 3 to 7).

**Table 3:** Post-TAVR Outcomes by BMI Group

	n	Underweight (n = 45)	Normal (n = 576)	Overweight (n = 438)	Obesity (n = 174)	Overall (N = 1,339)	*P* Value^a^	
							Unadjusted	Adjusted
30-d outcomes								
Mortality	1,283	0 (0)	10 (1.8)	10 (2.4)	5 (1.9)	25 (1.9)	0.72	0.81
Rehospitalization	1,265	1 (2.3)	50 (9.2)	37 (9.0)	22 (8.4)	110 (8.7)	0.49	0.33
Major and life-threatening bleeding	1,159	4 (10.3)	67 (13.6)	33 (8.7)	4 (1.6)	108 (9.3)	<0.001	0.40
Major vascular injury	1,255	0 (0.0)	19 (3.5)	11 (2.7)	6 (2.3)	36 (2.9)	0.49	>0.99
Stroke	1,262	1 (2.3)	17 (3.1)	10 (2.4)	5 (1.9)	33 (2.6)	0.78	0.85
Pacemaker implantation	1,255	4 (9.5)	42 (7.7)	51 (12.5)	35 (13.4)	132 (10.5)	0.039	0.36
Acute kidney injury stage 2 or 3	369	0 (0)	0 (0)	1 (0.8)	2 (1.5)	3 (0.8)	0.63	NA
1-outcomes								
Mortality	876	3 (10.3)	46 (12.4)	39 (13.7)	34 (17.9)	122 (13.9)	0.31	0.82
Rehospitalization	868	7 (24.1)	129 (34.3)	91 (32.4)	61 (33.5)	288 (33.2)	0.71	0.31
Major and life-threatening bleeding	732	4 (16.0)	69 (21.7)	35 (14.6)	5 (3.4)	113 (15.4)	<0.001	0.14
Major vascular injury	763	0 (0)	20 (6.1)	11 (4.4)	7 (4.4)	38 (5.0)	0.49	>0.99
Stroke	779	1 (3.7)	24 (7.1)	12 (4.7)	8 (5.0)	45 (5.8)	0.55	0.67
Pacemaker implantation	795	4 (15.4)	44 (13.0)	53 (20.0)	36 (21.7)	137 (17.2)	0.046	0.28
Acute kidney injury stage 2 or 3	308	0 (0)	0 (0)	1 (0.9)	2 (1.9)	3 (1.0)	0.60	NA

Values are n (%).

NA = non-applicable; other abbreviations as in Table 1.

a Unadjusted *P* value for differences across groups were compared by chi-square test. Adjusted *P* value from type III summary statistics of group in a multivariable logistic regression model adjusted for baseline characteristics of age, STS risk score, creatinine, hemoglobin, sex, race, coronary artery disease, atrial fibrillation, peripheral artery disease, diabetes mellitus, hypertension, preop pacemaker, bicuspid aortic disease, chronic kidney disease, end stage renal disease, NYHA functional class III/IV vs I/II, and use of general anesthesia.

Table 4 demonstrates results from 568 subjects with echocardiograms available at 1-year post-TAVR. Median change from baseline to 1-year in left ventricular ejection fraction was similar across groups (*P* = 0.08). Obese individuals experienced less median left ventricular mass index reduction (−5.1 g/m^2^; Q1-Q3: −26.9 to −16.2 g/m^2^) compared to underweight (−12.4 g/m^2^; Q1-Q3: −38.6 to −1.8 g/m^2^), normal weight (−19.5 g/m^2^; Q1-Q3: −41.6 to −3.6 g/m^2^), and overweight individuals (−12.6 g/m^2^; Q1-Q3: −34.2 to −4.4 g/m^2^; *P* < 0.001). Underweight individuals experienced greater residual tricuspid regurgitation (15% vs 3%; *P* = 0.054).

**Table 4:** 1-Year Post-TAVR Echocardiographic Outcomes

	n	Underweight (n = 20)	Normal (n = 257)	Overweight (n = 179)	Obesity (n = 112)	Overall (N = 568)	*P* Value^a^
Left ventricular ejection fraction, %	567	64 (51-69)	62 (57-66)	63 (56-66)	62 (57-67)	62 (57-66)	0.97
Change in left ventricular ejection fraction	567	2 (−6 to 10)	2 (−4 to 7)	0 (−5 to 6)	0 (−5 to 6)	1 (−4 to −7)	0.08
LVMI, g/m^2^	562	93 (81-112)	102 (86-120)	98 (81-121)	102 (83-125)	100 (83-121)	0.41
Change in LVMI, g/m^2^	560	−12.4 (−38.6 to −1.8)	−19.5 (−41.6 to −3.6)	−12.6 (−34.2 to −4.4)	−5.1 (−26.9 to −16.2)	−16.4 (−36.3 to −2.8)	<.001
Remodeling pattern	562						0.06
Normal		7 (35)	70 (27)	37 (21)	16 (15)	130 (23)	
Concentric hypertrophy		3 (15)	70 (27)	48 (27)	38 (35)	159 (28)	
Concentric remodeling		6 (30)	78 (30)	68 (39)	46 (42)	198 (35)	
Eccentric hypertrophy		4 (20)	38 (15)	23 (13)	10 (9)	75 (13)	
Peak velocity, m/s	566	2.1 (2.0-2.3)	2.3 (2.1-2.6)	2.4 (2.1-2.8)	2.5 (2.1-2.8)	2.4 (2.1-2.7)	0.052
Mean gradient, mm Hg	567	9 (7-11)	11 (9-15)	12 (9-16)	12 (8-17)	11 (9-15)	0.09
EROA, cm^2^	520	1.5 ± 0.6	1.4 ± 0.4	1.5 ± 0.4	1.6 ± 0.4	1.5 ± 0.4	0.042
Patient prosthetic mismatch	520	0 (0)	0 (0)	0 (0)	0 (0)	0 (0)	NA
Prosthetic valve stenosis	520	1 (5)	18 (8)	13 (8)	4 (4)	36 (7)	0.58
≥ Moderate AR	568	1 (5)	12 (5)	5 (3)	0 (0)	18 (3)	0.058
≥ Moderate MR	565	2 (10)	11 (4)	5 (3)	2 (2)	20 (4)	0.21
≥ Moderate TR	565	3 (15)	9 (4)	4 (2)	2 (2)	18 (3)	0.054

Values are mean ± SD, n (%), or median (Q1-Q3).

EROA = effective regurgitant orifice area; other abbreviations as in Tables 1 and 2.

a Differences across groups were compared by analysis of variance test, Kruskal-Wallis test, chi-square test, or Fisher exact test as appropriate.

## Discussion

In this contemporary, international, multicenter cohort of consecutive patients undergoing TAVR, the following key findings were observed:

There was no difference in the primary outcome of overall survival between BMI groups, even in subgroup analyses stratified by Asian and non-Asian patients.

Preprocedural BMI was associated with distinct risk factors. Underweight individuals were more likely to be older, have higher rates of CKD, and have a higher predicted risk of surgical aortic valve replacement mortality. Conversely, obese individuals were more likely to have both atherosclerotic risk factors and established cardiovascular disease.

Underweight individuals exhibited subtle differences in preprocedural echocardiographic markers, including evidence of adverse ventricular remodeling and greater aortic stenosis severity. However, post-TAVR, underweight patients demonstrated favorable echocardiographic improvements in left ventricular remodeling.

To the investigators' knowledge, this is one of the first studies to comprehensively evaluate the effect of pre-TAVR BMI on clinical and echocardiographic outcomes and the impact of post-TAVR BMI changes on long-term outcomes, with a unique focus on both Asian and non-Asian populations (Central Illustration).

**Central Illustration:**
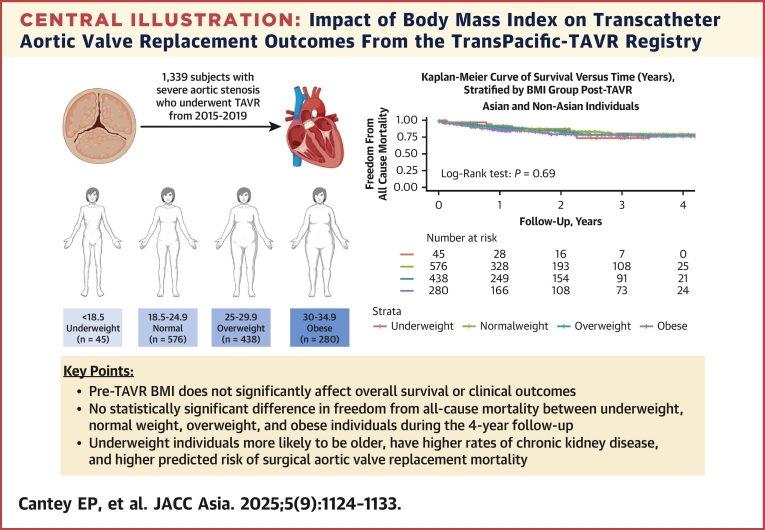
Impact of Body Mass Index on Transcatheter Aortic Valve Replacement Outcomes From the TransPacific-TAVR Registry This central illustration from the TP (TransPacific)-TAVR Registry examines the impact of body mass index (BMI) on transcatheter aortic valve replacement (TAVR) outcomes. We analyzed 1,339 patients with severe aortic stenosis who underwent TAVR (2015-2019), stratified by BMI. The Kaplan-Meier curve illustrates freedom from all-cause mortality across BMI groups post-TAVR. Results indicated no statistically significant difference in overall survival or clinical outcomes attributable to pre-TAVR BMI (log-rank test *P* = 0.69). Whereas BMI did not significantly affect survival, underweight individuals were noted to be older, have more chronic kidney disease, and have a higher predicted risk of surgical aortic valve replacement mortality.

Previous single-center studies have associated low BMI with decreased post-TAVR survival.^7^^,^^10^^,^^15^ Tezuka et al^16^ found similar 30-day mortality rates between BMI groups; however, the underweight group experienced worse midterm outcomes in Japanese patients. This was thought to be secondary to noncardiovascular factors, including a poorer nutritional baseline, as evidenced by low albumin being most significant, and respiratory disease.^16^ Conversely, the impact of obesity on long-term cardiovascular outcomes has been conflicting. Madanat et al^17^ performed a multicenter analysis that revealed a higher 30-day and 3-year survival rate following TAVR than in normal and underweight patients. Notably, this benefit did not extend beyond group I obesity.^17^ A large European registry identified a protective effect of elevated BMI on survival following TAVR.^9^ However, when a propensity-score analysis was performed to adjust for preprocedural comorbidities, Yamamoto et al^8^ found no significant difference in post-TAVR survival between BMI groups. This study corroborates those findings, demonstrating similar short- and long-term survival between underweight individuals and the rest of the cohort, even when stratified by race.

Our study observed that underweight patients, often with lower albumin and hemoglobin levels, exhibited distinct baseline characteristics and post-TAVR outcomes. Low BMI and nutritional deficiencies are crucial components of frailty, a well-established predictor of adverse outcomes following TAVR. Although our study did not directly assess frailty, the observed association among low BMI, potential nutritional deficiencies, and increased mortality with BMI decline emphasizes the importance of considering frailty in this population. Future studies should incorporate comprehensive frailty assessments, such as handgrip strength measurements and the Essential Frailty Toolset, to better risk-stratify patients and tailor interventions. Addressing frailty through targeted nutritional support and pre- and post-TAVR rehabilitation programs may improve outcomes in vulnerable patients. Given the high prevalence of underweight in the Asian cohort, future research should explore culturally tailored nutritional interventions to optimize outcomes in this population.

The "obesity paradox," observed in other cardiovascular conditions such as acute coronary syndrome and cardiac surgery, suggests a potential survival benefit for overweight or obese individuals.^18^, ^19^, ^20^, ^21^ However, this data, derived from a predominantly Asian population, does not support the presence of an obesity paradox in this contemporary TAVR cohort. Instead, the findings highlight the distinct characteristics of obese and underweight groups. Obese patients were more likely to have risk factors for atherosclerotic cardiovascular disease, such as hypertension, hyperlipidemia, and diabetes, as well as atherosclerotic cardiovascular disease itself. In contrast, underweight patients were older, more likely to have CKD, and had a higher predicted mortality risk. These unique, competing risks may have offset one another, resulting in no overall survival difference.

Underweight individuals exhibited markers of adverse pre-TAVR ventricular remodeling, more severe aortic stenosis, and higher rates of concomitant valvular disease. Underweight patients demonstrated greater regression in left ventricular mass, improved left ventricular systolic function, and comparable valvular hemodynamics at 1-year follow-up despite these baseline differences. Although the exact mechanisms underlying these observations warrant further investigation, we suspect that improved valve hemodynamics post-TAVR reduces afterload, enhances myocardial demand, and positively influences neurohormonal systems (eg, renin-angiotensin-aldosterone system) that contribute to left ventricular hypertrophy. We may also observe more pronounced reverse remodeling in patients with smaller ventricular cavities. However, these findings were not clinically significant, potentially due to the small sample size and limited follow-up duration. Although this may be influenced by selection bias, it suggests that favorable valvular function could improve valve-related outcomes over more extended follow-up periods. Future analyses should explore the relationship between these hemodynamic changes and clinically relevant outcomes such as functional capacity (eg, NYHA functional class), heart failure hospitalizations, and overall quality of life.

### Study limitations

This study has limitations inherent to retrospective, multicenter registries. Although using a standard database model and strict abstraction protocols enhances data integrity, the absence of frailty data and other confounding clinical conditions (eg, cancer or cirrhosis) remains a limitation. Nonetheless, the adequately powered, racially diverse, and contemporary cohort helps mitigate some of these concerns. Another notable limitation is that this study used World Health Organization BMI classifications, which may not fully account for differences in body composition and associated adiposity-related risks across ethnicities. Various organizations suggest that lower BMI thresholds may be more appropriate for defining overweight and obesity in Asian populations, with cutpoints of ≥23 kg/m^2^ and ≥25 kg/m^2^, respectively, proposed. The impact of these alternative cutoffs on our results was not explored and may represent a potential area for future study. Lastly, this analysis is not powered to assess potential interactions between balloon-expandable and self-expanding TAVR valves based on body weight.

## Conclusions

In contrast to prior investigations, pre-TAVR BMI was not associated with worse postprocedural survival or clinical outcomes in this cohort. Furthermore, underweight patients exhibited favorable post-TAVR hemodynamics and left ventricular remodeling; however, this study highlights the distinct characteristics of obese and underweight groups. Long-term studies should investigate the impact of nutritional and functional rehabilitation post-TAVR on clinical and echocardiographic outcomes, focusing on developing targeted interventions for underweight patients. Further research is also needed to determine the optimal strategies for risk stratification in diverse populations, potentially incorporating frailty assessments and alternative measures of body composition in addition to BMI.

## Funding Support and Author Disclosures

The Cardiovascular Research Foundation and the Asan Institute for Life Sciences and Corporate Relations partially funded the study but had no role in its design, data analysis, or manuscript preparation. The authors have reported that they have no relationships relevant to the contents of this paper to disclose.
