# Supplementary material for: Body Mass Index and Outcomes After Transcatheter Aortic Valve Replacement: Insights From the Transpacific-TAVR Registry
Source: JACC Asia. 2025 Sep 2;5(9):1124–33. doi: 10.1016/j.jacasi.2025.06.012 (PMC12426684; doi:10.1016/j.jacasi.2025.06.012)
Supplement: Supplemental Tables 1-7 [file mmc1.docx]

**Supplemental Table 1. Baseline characteristics by BMI group and Race**

| **Variable, No. (%)** | **Underweight** | | **Normal** | | **Overweight** | | **Obesity** | | **Overall** | | **P-value^1^** | |  |
| --- | --- | --- | --- | --- | --- | --- | --- | --- | --- | --- | --- | --- | --- |
|  | **Asian (n = 24)** | **Non-Asian (n = 21)** | **Asian (n = 341)** | **Non-Asian (n = 235)** | **Asian (n = 168)** | **Non-Asian (n = 270)** | **Asian (n = 28)** | **Non-Asian (n = 252)** | **Asian (n = 561)** | **Non-Asian (n = 778)** | **Asian** | **Non-Asian** | |
| Age at TAVR (years), mean ± SD | 82.6 ± 4.4 | 85.1 ± 8.3 | 80.5 ± 5.4 | 83.0 ± 8.6 | 79.3 ± 5.6 | 82.0 ± 8.2 | 80.7 ± 5.4 | 77.0 ± 8.5 | 80.2 ± 5.5 | 80.8 ± 8.8 | 0.013 | <.001 | |
| Male | 11 (45.8) | 6 (28.6) | 186 (54.5) | 115 (48.9) | 76 (45.2) | 165 (61.1) | 11 (39.3) | 139 (55.2) | 284 (50.6) | 425 (54.6) | 0.13 | 0.004 | |
| BMI (kg/m^2^), mean ± SD | 17.1 ± 1.1 | 16.8 ± 1.6 | 22.4 ± 1.7 | 22.5 ± 1.8 | 27.0 ± 1.4 | 27.5 ± 1.4 | 32.4 ± 4.0 | 35.9 ± 6.0 | 24.0 ± 3.6 | 28.4 ± 6.8 | <.001 | <.001 | |
| **Comorbidities** |  |  |  |  |  |  |  |  |  |  |  |  | |
| Coronary artery disease | 6 (25.0) | 19 (90.5) | 108 (31.7) | 180 (76.6) | 55 (32.7) | 208 (77.0) | 10 (35.7) | 190 (75.4) | 179 (31.9) | 597 (76.7) | 0.86 | 0.48 | |
| Myocardial infarction | 2 (8.3) | 6 (28.6) | 21 (6.2) | 38 (16.2) | 7 (4.2) | 45 (16.7) | 2 (7.1) | 30 (11.9) | 32 (5.7) | 119 (15.3) | 0.73 | 0.13 | |
| Percutaneous coronary intervention | 6 (25.0) | 7 (33.3) | 97 (28.4) | 70 (29.8) | 50 (29.8) | 80 (29.6) | 6 (21.4) | 77 (30.6) | 159 (28.3) | 234 (30.1) | 0.81 | 0.98 | |
| Atrial fibrillation or flutter | 2 (8.3) | 9 (42.9) | 42 (12.3) | 90 (38.3) | 18 (10.7) | 110 (40.7) | 5 (17.9) | 102 (40.5) | 67 (11.9) | 311 (40.0) | 0.68 | 0.93 | |
| Peripheral arterial disease | 0 (0) | 6 (28.6) | 14 (4.1) | 58 (24.7) | 4 (2.4) | 63 (23.3) | 1 (3.6) | 67 (26.6) | 19 (3.4) | 194 (24.9) | 0.59 | 0.83 | |
| Stroke | 3 (12.5) | 0 (0) | 47 (13.8) | 19 (8.1) | 22 (13.1) | 35 (13.0) | 1 (3.6) | 24 (9.5) | 73 (13.0) | 78 (10.0) | 0.50 | 0.11 | |
| Diabetes Mellitus | 8 (33.3) | 6 (28.6) | 167 (49.0) | 51 (21.7) | 99 (58.9) | 78 (28.9) | 21 (75.0) | 128 (50.8) | 295 (52.6) | 263 (33.8) | 0.003 | <.001 | |
| Diabetes Mellitus on Insulin | 0 (0) | 12 (57.1) | 23 (6.7) | 121 (51.5) | 12 (7.1) | 141 (52.2) | 6 (21.4) | 128 (50.8) | 41 (7.3) | 402 (51.7) | 0.016 | 0.95 | |
| Hypertension | 16 (66.7) | 18 (85.7) | 296 (86.8) | 193 (82.1) | 153 (91.1) | 231 (85.6) | 27 (96.4) | 223 (88.5) | 492 (87.7) | 665 (85.5) | 0.003 | 0.26 | |
| Hyperlipidemia | 10 (41.7) | 14 (66.7) | 257 (75.4) | 164 (69.8) | 129 (76.8) | 209 (77.4) | 27 (96.4) | 185 (73.4) | 423 (75.4) | 572 (73.5) | <.001 | 0.23 | |
| History of Pacemaker | 0 (0) | 3 (14.3) | 11 (3.2) | 24 (10.2) | 3 (1.8) | 16 (5.9) | 1 (3.6) | 28 (11.1) | 15 (2.7) | 71 (9.1) | 0.65 | 0.14 | |
| Bicuspid Aortic Valve | 4 (16.7) | 2 (9.5) | 34 (10.0) | 11 (4.7) | 19 (11.3) | 5 (1.9) | 0 (0) | 12 (4.8) | 57 (10.2) | 30 (3.9) | 0.21 | 0.13 | |
| Chronic Kidney Disease | 20 (83.3) | 10 (47.6) | 277 (81.2) | 97 (41.3) | 106 (63.1) | 96 (35.6) | 18 (64.3) | 83 (32.9) | 421 (75.0) | 286 (36.8) | <.001 | 0.18 | |
| End Stage Renal Disease on Dialysis | 0 (0) | 0 (0) | 16 (4.7) | 12 (5.1) | 5 (3.0) | 6 (2.2) | 0 (0) | 11 (4.4) | 21 (3.7) | 29 (3.7) | 0.37 | 0.26 | |
| NYHA Class III or IV | 12 (50.0) | 11 (52.4) | 121 (35.5) | 109 (46.4) | 55 (32.7) | 117 (43.3) | 12 (42.9) | 140 (55.6) | 200 (35.7) | 377 (48.5) | 0.33 | 0.038 | |
| STS Risk Score (%), median (IQR) | 4.9  (3.0-6.5) | 6.5  (4.9-10.4) | 3.5  (2.5-5.1) | 5.6  (3.6-8.7) | 2.8  (1.8-4.1) | 4.5  (3.1-6.5) | 3.5  (2.1-4.7) | 4.0  (3.0-6.2) | 3.3  (2.4-4.9) | 4.7  (3.2- 7.1) | <.001 | <.001 | |
| Creatinine (mg/dl), median (IQR) | 1.0  (0.8-1.2) | 0.9  (0.8-1.0) | 1.0  (0.8-1.2) | 1.0  (0.8-1.3) | 0.9  (0.8-1.1) | 1.1  (0.9-1.4) | 1.1  (0.8-1.4) | 1.1  (0.9-1.4) | 1.0  (0.8-1.2) | 1.0  (0.8-1.3) | 0.62 | 0.009 | |
| Albumin (mg/dl), median (IQR) | 3.5  (3.2-3.8) | 3.7  (3.6-4.3) | 3.5  (3.3-3.7) | 4.0  (3.7-4.2) | 3.6  (3.4-3.9) | 4.0  (3.8-4.3) | 3.6  (3.3-3.8) | 4.0  (3.8-4.2) | 3.5  (3.3-3.8) | 4.0  (3.8-4.2) | 0.09 | 0.53 | |
| Hemoglobin (g/dl), mean ± SD | 11.1 ± 1.7 | 11.4 ± 1.5 | 11.4 ± 1.9 | 12.0 ± 1.7 | 12.0 ± 1.9 | 12.3 ± 1.9 | 11.9 ± 1.8 | 12.3 ± 1.9 | 11.6 ± 1.9 | 12.2 ± 1.8 | 0.006 | 0.08 | |
| **Procedural Characteristics** |  |  |  |  |  |  |  |  |  |  |  |  | |
| Trans-femoral access | 22 (91.7) | 21 (100.0) | 330 (96.8) | 223 (94.9) | 164 (97.6) | 262 (97.0) | 26 (92.9) | 244 (96.8) | 542 (96.6) | 750 (96.4) | 0.31 | 0.43 | |
| Balloon-expandable valve | 22 (91.7) | 18 (85.7) | 287 (84.2) | 207 (88.1) | 144 (85.7) | 246 (91.1) | 21 (75.0) | 225 (89.3) | 474 (84.5) | 696 (89.5) | 0.38 | 0.67 | |
| General Anesthesia | 5 (20.8) | 11 (52.4) | 83 (24.3) | 137 (58.3) | 38 (22.6) | 140 (51.9) | 10 (35.7) | 125 (49.6) | 136 (24.2) | 413 (53.1) | 0.49 | 0.27 | |

^1^Differences across groups were compared by ANOVA test, Kruskal-Wallis test, or chi-squared test as appropriate.

**Supplemental Table 2. Baseline Echocardiographic Characteristics by Race**

| **Variable, n (%)** | **Underweight** | | **Normal** | | **Overweight** | | **Obesity** | | **Overall** | | **P-value** | |
| --- | --- | --- | --- | --- | --- | --- | --- | --- | --- | --- | --- | --- |
|  | **Asian (n = 24)** | **Non-Asian (n = 21)** | **Asian (n = 341)** | **Non-Asian (n = 235)** | **Asian (n = 168)** | **Non-Asian (n = 270)** | **Asian (n = 28)** | **Non-Asian (n = 252)** | **Asian (n = 561)** | **Non-Asian (n = 778)** | **Asian** | **Non-Asian** |
| Left Ventricular Ejection fraction (%), median (IQR) | 59  (47-66) | 58  (48-66) | 61  (55-65) | 58  (47-65) | 62  (56-65) | 60  (55-66) | 61  (55-64) | 62  (54-66) | 61  (56-65) | 60  (51-66) | 0.48 | 0.001 |
| Left Ventricular Ejection Fraction <40% | 5 (20.8) | 5 (23.8) | 42 (12.3) | 57 (24.3) | 12 (7.1) | 44 (16.3) | 1 (3.6) | 30 (11.9) | 60 (10.7) | 136 (17.5) | 0.07 | 0.003 |
| LVMI (g/m^2^), median (IQR) | 139  (107-151) | 100  (88-165) | 129  (108-154) | 105  (89-131) | 124  (108-144) | 108  (91-127) | 103  (91-150) | 106  (85-129) | 127  (107-151) | 106  (87-129) | 0.039 | 0.84 |
| Remodeling Pattern |  |  |  |  |  |  |  |  |  |  | 0.45 | 0.50 |
| Normal | 1 (4.2) | 0 (0.0) | 22 (6.8) | 10 (9.4) | 14 (8.8) | 14 (11.2) | 5 (21.7) | 15 (11.5) | 42 (7.9) | 39 (10.6) |  |  |
| Concentric Hypertrophy | 12 (50.0) | 1 (14.3) | 180 (55.7) | 43 (40.6) | 88 (55.3) | 49 (39.2) | 10 (43.5) | 49 (37.4) | 290 (54.8) | 142 (38.5) |  |  |
| Concentric Remodeling | 6 (25.0) | 4 (57.1) | 50 (15.5) | 39 (36.8) | 24 (15.1) | 50 (40.0) | 3 (13.0) | 58 (44.3) | 83 (15.7) | 151 (40.9) |  |  |
| Eccentric Hypertrophy | 5 (20.8) | 2 (28.6) | 71 (22.0) | 14 (13.2) | 33 (20.8) | 12 (9.6) | 5 (21.7) | 9 (6.9) | 114 (21.6) | 37 (10.0) |  |  |
| Peak velocity (m/s), mean ± SD | 4.9 ± 0.9 | 4.2 ± 0.8 | 4.8 ± 0.8 | 4.3 ± 0.7 | 4.8 ± 0.8 | 4.4 ± 0.7 | 4.5 ± 0.6 | 4.3 ± 0.6 | 4.8 ± 0.8 | 4.3 ± 0.7 | 0.33 | 0.30 |
| Mean gradient (mmHg), median (IQR) | 58  (42-70) | 41  (34-55) | 54  (42-70) | 43  (36-52) | 53  (43-69) | 44  (38-55) | 45  (41-60) | 45  (38-53) | 53  (42-70) | 44  (37-53) | 0.28 | 0.37 |
| AVA (cm^2^), mean ± SD | 0.57 ± 0.14 | 0.62 ± 0.21 | 0.61 ± 0.16 | 0.67 ± 0.19 | 0.62 ± 0.16 | 0.70 ± 0.19 | 0.62 ± 0.15 | 0.77 ± 0.19 | 0.61 ± 0.16 | 0.71 ± 0.19 | 0.46 | <.001 |
| Low Flow Low Gradient |  |  |  |  |  |  |  |  |  |  | 0.06 | 0.17 |
| None | 18 (75.0) | 14 (66.7) | 287 (84.2) | 143 (60.9) | 140 (83.3) | 182 (67.4) | 21 (75.0) | 180 (71.4) | 466 (83.1) | 519 (66.7) |  |  |
| Classical | 6 (25.0) | 5 (23.8) | 40 (11.7) | 65 (27.7) | 16 (9.5) | 63 (23.3) | 3 (10.7) | 43 (17.1) | 65 (11.6) | 176 (22.6) |  |  |
| Paradoxical | 0 (0) | 2 (9.5) | 14 (4.1) | 27 (11.5) | 12 (7.1) | 25 (9.3) | 4 (14.3) | 29 (11.5) | 30 (5.3) | 83 (10.7) |  |  |
| > moderate AR | 7 (29.2) | 6 (28.6) | 67 (19.6) | 22 (9.4) | 26 (15.5) | 19 (7.0) | 4 (14.3) | 17 (6.7) | 104 (18.5) | 64 (8.2) | 0.33 | 0.004 |
| > moderate MR | 5 (20.8) | 9 (42.9) | 46 (13.5) | 53 (22.6) | 12 (7.1) | 55 (20.4) | 2 (7.1) | 27 (10.7) | 65 (11.6) | 144 (18.5) | 0.07 | <.001 |
| > moderate TR | 2 (8.3) | 7 (33.3) | 22 (6.5) | 46 (19.6) | 10 (6.0) | 40 (14.8) | 2 (7.1) | 23 (9.1) | 36 (6.4) | 116 (14.9) | 0.97 | 0.001 |

^1^Differences across groups were compared by ANOVA test, Kruskal-Wallis test, or chi-squared test as appropriate.

**Supplemental Table 3. Post-TAVR outcomes by BMI group and by Race**

| **Variable, n (%)** | **N** | | **Underweight** | | **Normal** | | **Overweight** | | **Obesity** | | **Overall** | | **P-value^1^** | |
| --- | --- | --- | --- | --- | --- | --- | --- | --- | --- | --- | --- | --- | --- | --- |
|  | **Asian** | **Non-Asian** | **Asian (n = 24)** | **Non-Asian (n = 21)** | **Asian (n = 341)** | **Non-Asian (n = 235)** | **Asian (n = 168)** | **Non-Asian (n = 270)** | **Asian (n = 28)** | **Non-Asian (n = 252)** | **Asian (n = 561)** | **Non-Asian (n = 778)** | **Asian** | **Non-Asian** |
| **30-Day Outcomes** |  |  |  |  |  |  |  |  |  |  |  |  |  |  |
| Mortality | 551 | 732 | 0 (0) | 0 (0) | 4 (1.2) | 6 (2.7) | 4 (2.4) | 6 (2.4) | 0 (0) | 5 (2.1) | 8 (1.5) | 17 (2.3) | 0.58 | 0.88 |
| Rehospitalization | 544 | 721 | 0 (0) | 1 (5.3) | 27 (8.2) | 23 (10.7) | 9 (5.6) | 28 (11.2) | 1 (3.7) | 21 (8.9) | 37 (6.8) | 73 (10.1) | 0.32 | 0.74 |
| Major and life-threatening bleeding | 469 | 690 | 4 (19.0) | 0 (0) | 64 (22.4) | 3 (1.5) | 28 (20.0) | 5 (2.1) | 3 (13.6) | 1 (0.4) | 99 (21.1) | 9 (1.3) | 0.76 | 0.43 |
| Major Vascular injury | 545 | 710 | 0 (0) | 0 (0) | 15 (4.5) | 4 (1.9) | 7 (4.3) | 4 (1.6) | 2 (7.4) | 4 (1.7) | 24 (4.4) | 12 (1.7) | 0.64 | 0.95 |
| Stroke | 545 | 717 | 1 (4.2) | 0 (0) | 11 (3.3) | 6 (2.8) | 3 (1.9) | 7 (2.8) | 1 (3.7) | 4 (1.7) | 16 (2.9) | 17 (2.4) | 0.80 | 0.72 |
| Pacemaker implantation | 544 | 711 | 3 (12.5) | 1 (5.6) | 20 (6.0) | 22 (10.4) | 13 (8.0) | 38 (15.4) | 2 (7.4) | 33 (14.0) | 38 (7.0) | 94 (13.2) | 0.60 | 0.32 |
| Acute Kidney Injury Stage 2 or 3 | 5 | 364 | 0 (0) | 0 (0) | 0 (0) | 0 (0) | 0 (0) | 1 (0.8) | 0 (0) | 2 (1.5) | 0 (0) | 3 (0.8) | NA | 0.63 |
| **1-Year Outcomes** |  |  |  |  |  |  |  |  |  |  |  |  |  |  |
| Mortality | 375 | 501 | 1 (5.6) | 2 (18.2) | 24 (10.5) | 22 (15.3) | 12 (10.9) | 27 (15.4) | 0 (0) | 34 (19.9) | 37 (9.9) | 85 (17.0) | 0.44 | 0.65 |
| Rehospitalization | 380 | 488 | 4 (22.2) | 3 (27.3) | 71 (30.2) | 58 (41.1) | 28 (26.2) | 63 (36.2) | 4 (20.0) | 57 (35.2) | 107 (28.2) | 181 (37.1) | 0.64 | 0.63 |
| Major and life-threatening bleeding | 334 | 398 | 4 (23.5) | 0 (0) | 66 (32.5) | 3 (2.6) | 29 (30.2) | 6 (4.2) | 3 (16.7) | 2 (1.5) | 102 (30.5) | 11 (2.8) | 0.49 | 0.56 |
| Major Vascular injury | 347 | 416 | 0 (0) | 0 (0) | 16 (7.7) | 4 (3.3) | 7 (6.9) | 4 (2.7) | 2 (10.5) | 5 (3.6) | 25 (7.2) | 13 (3.1) | 0.63 | 0.92 |
| Stroke | 352 | 427 | 1 (5.6) | 0 (0) | 17 (8.1) | 7 (5.6) | 5 (4.9) | 7 (4.6) | 1 (5.0) | 7 (5.0) | 24 (6.8) | 21 (4.9) | 0.73 | 0.89 |
| Pacemaker implantation | 352 | 443 | 3 (16.7) | 1 (12.5) | 22 (10.4) | 22 (17.5) | 14 (13.7) | 39 (23.9) | 2 (10.0) | 34 (23.3) | 41 (11.6) | 96 (21.7) | 0.74 | 0.49 |
| Acute Kidney Injury Stage 2 or 3 | 3 | 305 | 0 (0) | 0 (0) | 0 (0) | 0 (0) | 0 (0) | 1 (0.9) | 0 (0) | 2 (1.9) | 0 (0) | 3 (1.0) | NA | 0.60 |

^1^Differences across groups were compared by chi-squared test.

**Supplemental Table 4. Selection of covariates for multivariable modeling**

| **Variable, n (%)** | **N** | **P-value^1^** | | | **Multivariable Model Inclusion** |
| --- | --- | --- | --- | --- | --- |
|  |  | **Obesity Group** | **30-Day Mortality** | **1-Year Mortality** |  |
| Age at TAVR (years), mean ± SD | 1339 | <0.001 | 0.40 | 0.14 | Yes |
| Male | 1339 | 0.17 | 0.77 | 0.11 | Yes |
| Race | 1339 | <0.001 | 0.27 | 0.003 | Yes |
| BMI (kg/m^2^), mean ± SD | 1339 | <0.001 | 0.54 | 0.12 | No – Obesity Group as the primary predictor |
| Coronary artery disease | 1339 | <0.001 | 0.75 | 0.008 | Yes |
| Atrial fibrillation or flutter | 1339 | <0.001 | 0.16 | <0.001 | Yes |
| Peripheral arterial disease | 1339 | <0.001 | 0.24 | <0.001 | Yes |
| Diabetes Mellitus | 1339 | <0.001 | 0.53 | 0.002 | Yes |
| Diabetes Mellitus on Insulin | 1339 | <0.001 | 0.44 | <0.001 | No – due to collinearity with DM |
| Hypertension | 1339 | 0.042 | 0.18 | 0.09 | Yes |
| Hyperlipidemia | 1339 | 0.005 | 0.28 | 0.55 | No – due to non-significance |
| History of Pacemaker | 1339 | 0.015 | 0.74 | 0.029 | Yes |
| Bicuspid Aortic Valve | 1339 | 0.044 | 0.75 | 0.046 | Yes |
| Chronic Kidney Disease | 1339 | <0.001 | 0.46 | 0.11 | Yes |
| End Stage Renal disease on Dialysis | 1339 | 0.13 | 0.037 | <0.001 | Yes |
| NYHA Class III or IV | 1339 | <0.001 | 0.046 | 0.001 | Yes |
| STS Risk Score (%), mean ± SD | 1339 | <0.001 | 0.002 | <0.001 | Yes |
| Creatinine (mg/dl), median (IQR) | 1339 | 0.008 | 0.21 | 0.004 | Yes |
| Albumin (mg/dl), median (IQR) | 878 | <0.001 | 0.20 | 0.002 | No – due to sample size |
| Hemoglobin (g/dl), mean ± SD | 1339 | <0.001 | 0.52 | 0.054 | Yes |
| General Anesthesia | 1339 | 0.037 | 0.40 | <0.001 | Yes |

^1^P-value for association with obesity group is based on ANOVA, Kruskal-Wallis, or Chi-squared test. P-value for association with 30-day and 1-year mortality was based on univariate logistic regression.

**Supplemental Table 5. Baseline characteristics by BMI group**

| **Variable, n (%)** | **Normal (n = 576)** | **Overweight (n = 438)** | **Obesity (n = 280)** | **P-value^1^** |
| --- | --- | --- | --- | --- |
| Age at TAVR (years), mean ± SD | 81.5 ± 7.0 | 81.0 ± 7.4 | 77.4 ± 8.3 | <0.001 |
| Male | 301 (52.3) | 241 (55.0) | 150 (53.6) | 0.68 |
| Race |  |  |  |  |
| Non-Asian | 235 (40.8) | 270 (61.6) | 252 (90.0) | <0.001 |
| Asian | 341 (59.2) | 168 (38.4) | 28 (10.0) | <0.001 |
| BMI (kg/m^2^), mean ± SD | 22.4 ± 1.7 | 27.3 ± 1.4 | 35.5 ± 5.9 | <0.001 |
| **Comorbidities** |  |  |  |  |
| Coronary artery disease | 288 (50.0) | 263 (60.0) | 200 (71.4) | <0.001 |
| Myocardial infarction | 59 (10.2) | 52 (11.9) | 32 (11.4) | 0.70 |
| Percutaneous coronary intervention | 167 (29.0) | 130 (29.7) | 83 (29.6) | 0.97 |
| Atrial fibrillation or flutter | 132 (22.9) | 128 (29.2) | 107 (38.2) | <0.001 |
| Peripheral arterial disease | 72 (12.5) | 67 (15.3) | 68 (24.3) | <0.001 |
| Stroke | 66 (11.5) | 57 (13.0) | 25 (8.9) | 0.24 |
| Diabetes Mellitus | 218 (37.8) | 177 (40.4) | 149 (53.2) | <0.001 |
| Diabetes Mellitus on Insulin | 144 (25.0) | 153 (34.9) | 134 (47.9) | <0.001 |
| Hypertension | 489 (84.9) | 384 (87.7) | 250 (89.3) | 0.16 |
| Hyperlipidemia | 421 (73.1) | 338 (77.2) | 212 (75.7) | 0.32 |
| History of Pacemaker | 35 (6.1) | 19 (4.3) | 29 (10.4) | 0.005 |
| Bicuspid Aortic Valve | 45 (7.8) | 24 (5.5) | 12 (4.3) | 0.09 |
| Chronic Kidney Disease | 374 (64.9) | 202 (46.1) | 101 (36.1) | <0.001 |
| End Stage Renal disease on Dialysis | 28 (4.9) | 11 (2.5) | 11 (3.9) | 0.16 |
| NYHA Class III or IV | 230 (39.9) | 172 (39.3) | 152 (54.3) | <0.001 |
| STS Risk Score (%), median (IQR) | 4.0 (2.8-6.6) | 3.7 (2.6-5.8) | 4.0 (2.9-6.0) | 0.019 |
| Creatinine (mg/dl), median (IQR) | 1.0 (0.8-1.3) | 1.0 (0.8-1.3) | 1.1 (0.8-1.4) | 0.027 |
| Albumin (mg/dl), median (IQR) | 3.6 (3.3-3.9) | 3.8 (3.5-4.0) | 3.9 (3.7-4.2) | <0.001 |
| Hemoglobin (g/dl), mean ± SD | 11.7 ± 1.9 | 12.2 ± 1.9 | 12.2 ± 1.9 | <0.001 |
| **Procedural Characteristics** |  |  |  |  |
| Transfemoral access | 553 (96.0) | 426 (97.3) | 270 (96.4) | 0.56 |
| Balloon-expandable valve | 494 (85.8) | 390 (89.0) | 246 (87.9) | 0.29 |
| General Anesthesia | 220 (38.2) | 178 (40.6) | 135 (48.2) | 0.019 |

^1^Differences across groups were compared by ANOVA test, Kruskal-Wallis test, or chi-squared test as appropriate.

**Supplemental Table 6. Baseline echocardiographic characteristics by BMI group**

| **Variable, n (%)** | **Normal (n = 576)** | **Overweight (n = 438)** | **Obesity (n = 280)** | **P-value^1^** |
| --- | --- | --- | --- | --- |
| Left Ventricular Ejection fraction (%), median (IQR) | 60 (50-65) | 61 (55-65) | 62 (55-66) | 0.011 |
| Left Ventricular Ejection Fraction <40% | 99 (17.2) | 56 (12.8) | 31 (11.1) | 0.029 |
| LVMI (g/m^2^), median (IQR) | 125 (103-150) | 117 (100-136) | 105 (85-129) | <0.001 |
| Remodeling Pattern^^^ |  |  |  | <0.001 |
| Normal | 32 (7.5) | 28 (9.9) | 20 (13.0) |  |
| Concentric Hypertrophy | 223 (52) | 137 (48.2) | 59 (38.3) |  |
| Concentric Remodeling | 89 (20.7) | 74 (26.1) | 61 (39.6) |  |
| Eccentric Hypertrophy | 85 (19.8) | 45 (15.8) | 14 (9.1) |  |
| Peak velocity (m/s), mean ± SD | 4.6 ± 0.8 | 4.6 ± 0.8 | 4.3 ± 0.6 | <0.001 |
| Mean gradient (mmHg), median (IQR) | 48 (40-62) | 47 (40-60) | 45 (38-54) | <0.001 |
| AVA (cm^2^), mean ± SD | 0.63 ± 0.18 | 0.67 ± 0.18 | 0.75 ± 0.19 | <0.001 |
| Low Flow Low Gradient |  |  |  | 0.25 |
| None | 430 (74.7) | 322 (73.5) | 201 (71.8) |  |
| Classical | 105 (18.2) | 79 (18.0) | 46 (16.4) |  |
| Paradoxical | 41 (7.1) | 37 (8.4) | 33 (11.8) |  |
| > moderate AR | 89 (15.5) | 45 (10.3) | 21 (7.5) | 0.001 |
| > moderate MR | 99 (17.2) | 67 (15.3) | 29 (10.4) | 0.032 |
| > moderate TR | 68 (11.8) | 50 (11.4) | 25 (8.9) | 0.43 |

^1^Differences across groups were compared by ANOVA test, Kruskal-Wallis test, or chi-squared test as appropriate.

**Supplemental Table 7. Post-TAVR outcomes by BMI group**

| **Outcome, n (%)** | **N** | **Normal (n = 576)** | **Overweight (n = 438)** | **Obesity (n = 174)** | **P-value^1^** |
| --- | --- | --- | --- | --- | --- |
| **30-Day Outcomes** |  |  |  |  |  |
| Mortality | 1240 | 10 (1.8) | 10 (2.4) | 5 (1.9) | 0.80 |
| Rehospitalization | 1222 | 50 (9.2) | 37 (9.0) | 22 (8.4) | 0.93 |
| Major and life-threatening bleeding | 1120 | 67 (13.6) | 33 (8.7) | 4 (1.6) | <0.001 |
| Major Vascular injury | 1213 | 19 (3.5) | 11 (2.7) | 6 (2.3) | 0.58 |
| Stroke | 1219 | 17 (3.1) | 10 (2.4) | 5 (1.9) | 0.58 |
| Pacemaker implantation | 1213 | 42 (7.7) | 51 (12.5) | 35 (13.4) | 0.016 |
| Acute Kidney Injury Stage 2 or 3 | 360 | 0 (0) | 1 (0.8) | 2 (1.5) | 0.44 |
| **1-Year Outcomes** |  |  |  |  |  |
| Mortality | 847 | 46 (12.4) | 39 (13.7) | 34 (17.9) | 0.20 |
| Rehospitalization | 839 | 129 (34.3) | 91 (32.4) | 61 (33.5) | 0.87 |
| Major and life-threatening bleeding | 707 | 69 (21.7) | 35 (14.6) | 5 (3.4) | <0.001 |
| Major Vascular injury | 738 | 20 (6.1) | 11 (4.4) | 7 (4.4) | 0.59 |
| Stroke | 752 | 24 (7.1) | 12 (4.7) | 8 (5.0) | 0.40 |
| Pacemaker implantation | 769 | 44 (13.0) | 53 (20.0) | 36 (21.7) | 0.019 |
| Acute Kidney Injury Stage 2 or 3 | 302 | 0 (0) | 1 (0.9) | 2 (1.9) | 0.41 |

^1^Differences across groups were compared by chi-squared test.
